# Structure–function relationships explain CTCF zinc finger mutation phenotypes in cancer

**DOI:** 10.1007/s00018-021-03946-z

**Published:** 2021-10-16

**Authors:** Charles G. Bailey, Shailendra Gupta, Cynthia Metierre, Punkaja M. S. Amarasekera, Patrick O’Young, Wunna Kyaw, Tatyana Laletin, Habib Francis, Crystal Semaan, Mehdi Sharifi Tabar, Krishna P. Singh, Charles G. Mullighan, Olaf Wolkenhauer, Ulf Schmitz, John E. J. Rasko

**Affiliations:** 1 Cancer and Gene Regulation Laboratory Centenary Institute, The University of Sydney, Camperdown, NSW 2050 Australia; 2 Gene and Stem Cell Therapy Program Centenary Institute, The University of Sydney, Camperdown, NSW 2050 Australia; 3 Faculty of Medicine and Health, The University of Sydney, Sydney, NSW 2006 Australia; 4 Department of Systems Biology and Bioinformatics, University of Rostock, 18051 Rostock, Germany; 5 Chhattisgarh Swami Vivekanand Technical University, Bhilai, Chhattisgarh 491107 India; 6 Department of Pathology and Hematological Malignancies Program, St. Jude Children’s Research Hospital, Memphis, TN 38105-3678 USA; 7 Stellenbosch Institute for Advanced Study (STIAS), Wallenberg Research Centre at Stellenbosch University, Stellenbosch, South Africa; 8 Computational Biomedicine Centenary Institute, The University of Sydney, Camperdown, NSW 2050 Australia; 9 Cell and Molecular Therapies, Royal Prince Alfred Hospital, Camperdown, NSW 2050 Australia

**Keywords:** CTCF, Cancer, Zinc finger, Somatic mutation, Gain-of-function, Loss-of-function, Molecular docking, Molecular dynamics

## Abstract

CCCTC-binding factor (CTCF) plays fundamental roles in transcriptional regulation and chromatin architecture maintenance. CTCF is also a tumour suppressor frequently mutated in cancer, however, the structural and functional impact of mutations have not been examined. We performed molecular and structural characterisation of five cancer-specific CTCF missense zinc finger (ZF) mutations occurring within key intra- and inter-ZF residues. Functional characterisation of CTCF ZF mutations revealed a complete (L309P, R339W, R377H) or intermediate (R339Q) abrogation as well as an enhancement (G420D) of the anti-proliferative effects of CTCF. DNA binding at select sites was disrupted and transcriptional regulatory activities abrogated. Molecular docking and molecular dynamics confirmed that mutations in residues specifically contacting DNA bases or backbone exhibited loss of DNA binding. However, R339Q and G420D were stabilised by the formation of new primary DNA bonds, contributing to gain-of-function. Our data confirm that a spectrum of loss-, change- and gain-of-function impacts on CTCF zinc fingers are observed in cell growth regulation and gene regulatory activities. Hence, diverse cellular phenotypes of mutant CTCF are clearly explained by examining structure–function relationships.

**Supplementary Information:**

The online version contains supplementary material available at 10.1007/s00018-021-03946-z.

## Background

Comprehensive catalogues of somatic mutations have been assembled from surveying the genomic landscape in numerous human cancers. More than 200 large-scale studies involving cancer types or subtypes of clinical or societal importance have been deposited in the cBio Cancer Genome Portal [1]. These studies have provided new insights into cancer causation and offered new leads for potential therapeutic intervention using a genomics-driven oncology approach. Cancers are remarkably heterogeneous in their distribution and frequency of somatic mutations. Paediatric cancers contain as few as 0.1 mutations per megabase (Mb), whereas lung and melanoma samples may accumulate over 100/Mb (average 4.0/Mb) [2]. Whilst some genes are mutated at high frequencies, most genes are mutated at intermediate frequencies (2–20%) [3] adding to the complex molecular landscape underlying tumour biology. Those genes that exhibit mutation frequencies above background have been called significantly mutated genes, of which 127 have been identified amongst a dozen cancers [4]. These mutations disrupt diverse cellular processes including transcriptional regulation, histone modification, genome integrity, signalling and splicing [4]. A similar concept is cancer driver genes or driver mutations of which nearly 300 have been identified. Here, activation of oncogenes, or mutation or inactivation of tumour suppressor genes can cause a selective growth advantage in a direct or indirect manner [5, 6].

In tumour cells, recurrent acquired mutations have been observed in nearly every DNA, RNA and protein component of normal transcriptional control [7]. These somatic mutations may directly impact transcription factors (TFs) or indirectly via TF target sites in *cis*- and *trans*-regulatory elements, as well as chromatin architecture leading to transcriptional dysregulation in cancer. Dysregulation of transcriptional programs in cancer cells can lead to transcriptional dependencies that offer opportunities for exploitation with targeted therapeutic strategies [7]. For example, pharmacological inhibition of the BET bromodomain-containing BRD4 protein has emerged as a promising therapeutic strategy to prevent MYC-dependent transcriptional signaling in various haemopoietic malignancies [8–11]. Investigating and exploiting these acquired cellular vulnerabilities is a major thrust of many cancer research efforts.

Approximately 1600–2000 TFs have been validated or predicted within the human genome [12, 13]. TFs containing the zinc-coordinating C2H2 class of DNA binding domains represent the largest class of transcription factors [13], comprising nearly 50% of all TFs [14]. Human C2H2 TFs contain an average of ~ 10 zinc fingers (ZFs), specifying target sites of ~ 30 bases [15], however, not all ZFs contact DNA simultaneously or indeed, are involved in DNA binding. Furthermore, the functional impact of somatic mutations on many TFs is unknown. Nor is it known whether such changes impact DNA binding or transcriptional activation globally or in a locus-specific manner.

One such C2H2 ZF-containing transcription factor, CCCTC-binding factor (CTCF), features a central tandem array of 11 ZFs enabling multivalent binding to DNA target sites. Careful mutational analysis of key residues co-ordinating Zn^2+^ ion binding and ZF formation have shown key central ZFs that contribute binding to a core consensus site, whilst peripheral ZFs stabilise CTCF binding and bind additional conserved and non-conserved motifs [16]. Through combinatorial DNA binding and ZF multivalency, CTCF plays diverse roles in transcriptional regulation, including the regulation of alternative splicing (as recently reviewed in [17]). In its role co-ordinating three-dimensional genome architecture, CTCF has been named the ‘master weaver’ protein [18]. Unprecedented insights into the nuclear organisation, obtained from high-resolution conformational maps of chromatin interactions, have defined the rules governing CTCF-mediated chromatin organisation. First, CTCF links gene regulation to genomic architecture by co-ordinating DNA looping together with cohesin [19–21]. Second, CTCF defines the boundaries of topologically associating domains (TADs) [22–24] in a structural framework that is evolutionarily conserved [25]. Depletion of CTCF can result in loss of DNA looping and insulation within TADs, however, genomic compartmentalisation is preserved [26]. Finally, TAD organisation is CTCF site orientation-specific [25, 27], such that rewiring or inverting CTCF sites can significantly perturb gene expression by affecting promoter–enhancer interactions or disrupt discrete insulated territories during development [28–30].

CTCF plays an integral role in cell-type-specific genomic organisation and development. CTCF’s role in development and differentiation has been examined in at least seven tissues or developmental stages in mice, as well as zebrafish and Drosophila [31]. CTCF is absolutely essential, as *CTCF* null embryos are unable to implant [32] and maintenance of CTCF expression ensures somatic cell viability [33]. Extensive characterisation of the action of CTCF in vitro and in vivo has led to its classification as a haploinsufficient tumour suppressor gene [33–35]. Whilst isolated somatic CTCF mutations were first identified in some solid tumours [36], numerous cancer genome studies since have highlighted the impact and prevalence of CTCF mutation in multiple cancers [4, 5]. CTCF is a significantly mutated gene in ~ 20% of endometrial cancers [37–40] and is recurrently mutated in myeloid and lymphoid malignancies [41–44].

Despite many CTCF mutations having been identified in numerous cancer types, the functional consequences of these mutations have not been thoroughly examined. In this study, we performed a meta-analysis of all publicly available cancer mutation data for CTCF and showed a significant enrichment of missense mutations occurring in CTCF’s ZF DNA binding domain. We have functionally characterised a subset of representative ZF mutations detected in acute lymphoblastic leukaemia samples to examine their consequences. Finally, we compared the impact of CTCF ZF mutation on DNA binding, transcriptional activation as well as on CTCF ZF domain structure using molecular modelling and molecular dynamics simulations. This is the first study to examine the effect of somatic mutation on CTCF ZF structure–function relationships.

## Results

### CTCF ZF domain is enriched for somatic missense mutations in cancer

**Fig. 1:**
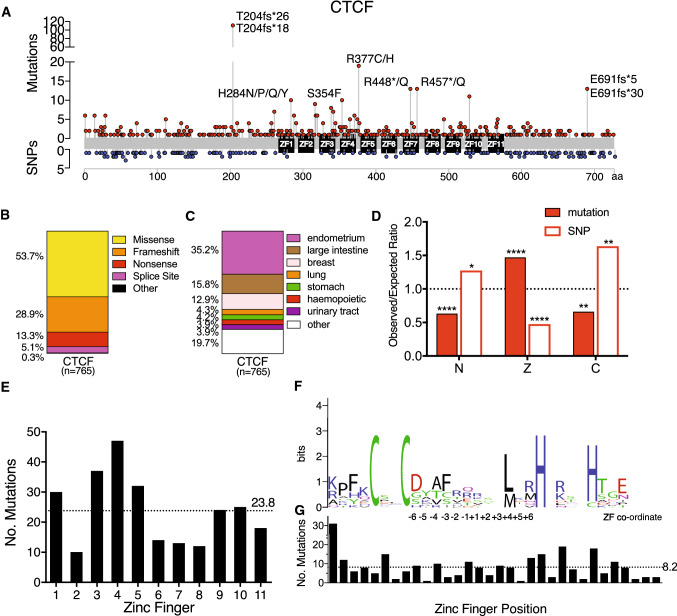
Distribution and impact of CTCF somatic mutations in cancer. **A** The landscape of somatic mutations (above) and SNPs (below) occurring in CTCF: the distribution and frequency within the coding region is shown, recurrent somatic mutations (occurring $\ge$ 10 times) are labelled. For a curated list of non-redundant CTCF mutations from cancer genome sequencing studies (TCGA, COSMIC) and published studies see Supplementary Table 1. *CTCF* mutation type (**B**); and tissue distribution (**C**) are shown; *n* = total number of mutations. **D** Analysis of cancer-related somatic missense variants and missense SNPs occurring in each domain of CTCF (N=N-terminus; Z=ZF domain; C=C-terminus). The expected occurrence was calculated from the total number with the proportion of missense variants expected in each domain if they were evenly distributed. The observed/expected ratio confirms if there is a de-enrichment (< 1.0) or an enrichment (> 1.0) of non-synonymous changes. **E** Frequency of somatic missense mutations occurring in specific ZFs of CTCF, the mean for all ZFs is shown (dotted line). **F** Sequence logo of all 11 aligned CTCF ZFs; numbers (− 6 to + 6) indicate co-ordinates within the DNA-binding portion of the ZF. Similar amino acids are coloured: black—hydrophobic (G, A, V, I, L, P, W, F, M); green—polar (S, T, Y, C); purple—polar amide (Q, N); blue—basic (K, R, H); and red—acidic (D, E). The height of each amino acid residue is proportional to its observed frequency. The overall height of each letter ‘stack’ is proportional to the sequence conservation, shown in bits. **G** Frequency of missense somatic mutations at each ZF position; the mean for all ZFs is shown (dotted line). Data represent the mean ± SD with statistical analysis performed using the Chi-square test (*p < 0.05; **p < 0.01; ****p < 0.0001)

We analysed cancer genome sequencing databases and published mutation data to determine the distribution, frequency and nature of somatic mutations occurring in CTCF (Supplementary Table 1). The distribution and frequency of all known somatic mutations in CTCF is shown with recurrent mutant residues indicated (Fig. 1A). The recurrent T204fs*26 and T204fs*18 mutations in CTCF arise due to a high frequency of insertions or deletions within a 30 bp purine-rich (> 85%) region at c.1048 –c.1077 encoding T204. Frequently occurring missense or nonsense mutations occur at H284, S354, R377, R448 and R457 within the ZF region of CTCF (Fig. 1A). Further analysis revealed that inactivating nonsense and frameshift mutations account for ~ 40% of somatic CTCF mutations (Fig. 1B). This result exceeds the ‘20/20 rule’ for tumour suppressor gene classification which requires that > 20% of somatic mutations are inactivating [6] and affirms our earlier work demonstrating CTCF’s role as a tumour suppressor [33–35]. CTCF mutations occur prominently in cancers arising in the endometrium and breast (~ 48%) (Fig. 1C), consistent with mutant CTCF being classified as a pan-gynaecological driver of cancer [5].

We next examined somatic missense mutations and SNPs reported for CTCF and compared their observed and expected occurrences (Supplementary Table 2). CTCF’s ZF domain has a significant enrichment for somatic missense mutations observed over the number expected for its relative size, such that the observed/expected (*O*/*E*) ratio = 1.47, (*p* < 0.0001). Furthermore, there was a de-enrichment of non-synonymous SNPs occurring within the ZF domain (*O*/*E* = 0.48, *p* < 0.0001) (Fig. 1D, Supplementary Table 2). These results suggest that the human CTCF ZF region is intolerant to normal genetic variation, but is frequently inactivated in cancer. As ZF mutations would likely affect DNA binding, these are likely to have a significant impact on CTCF function. There is a concomitant paucity of missense somatic mutations within the N- and C-termini of CTCF (*O*/*E* = 0.63, *p* < 0.0001 and *O*/*E*  = 0.65, *p* = 0.0269, respectively, Fig. 1D, Supplementary Table 2). Strikingly, the opposite pattern is observed for SNPs in CTCF with an enrichment of missense SNPs in the N-terminus (*O*/*E*  = 1.27, *p* = 0.0269) and C-terminus (O/E = 1.63, *p* = 0.0032) Fig. 1D, Supplementary Table 2). We then determined the potential functional impact of somatic missense mutations in CTCF using Polyphen analysis. Missense mutations exhibited an overall greater functional impact than missense SNPs (0.80 ± 0.35 vs 0.49 ± 0.44, mean ± SD, *p* < 0.0001, Supplementary Fig. 1A). Further analysis indicated that there was a decrease in the ratio of transition to transversion mutations when comparing SNPs to missense somatic mutations (2.24 to 1.19, respectively, *p* < 0.0001, Supplementary Fig. 1B). These data provide further support for the role of CTCF as a tumour suppressor that is frequently mutated and functionally impacted in cancer.

As the majority of somatic missense mutations in CTCF occur within the ZF domain we next analysed the distribution of missense mutations in specific ZFs of CTCF. We found that the greatest proportion of mutations occurred in ZF4 (~ 20%), followed by ZF3 (~ 15%) (Fig. 1E). ZFs 3–7 have been shown to be responsible for binding CTCF’s core 15 bp consensus, with other ZFs providing binding specificity depending on adjacent motifs [16, 45]. A sequence logo depicting all 11 ZFs in CTCF (10 C2H2- and 1 C2HC-type) shows the conserved Cys and His residues that co-ordinate Zn^2+^ binding, an invariant hydrophobic Leu or Met residue at + 4 and substantial amino acid variation at other positions (Fig. 1F). The proportion of mutations occurring at each position within ZFs was determined. This analysis revealed that the proportion of inter-ZF mutations was 31.5%, Cys/His mutations (17.7%) and those affecting key DNA binding residues (− 1, + 2, + 3, + 6, 15.6%). Thus, approximately one-third of missense CTCF ZF mutations have an unknown impact but likely affect ZF folding and stability.

### CTCF ZF mutations exhibit loss- and gain-of-function in cell growth phenotypes in vitro

**Fig. 2:**
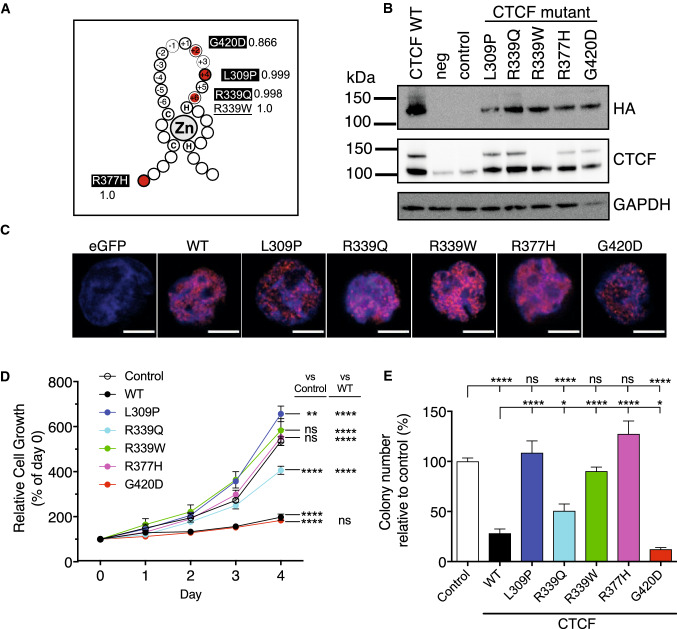
Functional impacts of CTCF ZF missense mutations. **A** Published and unpublished missense CTCF mutations (red circles) occurring in acute lymphoblastic leukaemia (L309P, R339Q, R337H, G420D—highlighted in black) superimposed on a C2H2 ZF structure: C = cysteine, H = histidine, Zn = Zn^2+^ ion; R339W (underlined) is a previously characterised change-of-function mutation used as a control. The Polyphen score for each mutation is indicated. Numbers (− 6 to + 6) indicate co-ordinates within the DNA-binding portion of the ZF; residues directly contacting DNA at positions − 1, + 2, + 3 and + 6 are indicated (white ring). **B** Western blot of WT and mutant CTCF expression in transduced K562 cells; anti-HA antibody detects ectopic CTCF, CTCF antibody detects total CTCF; GAPDH is a loading control; size markers indicate MW in kDa. **C** Immunofluorescence of HA-tagged WT and mutant CTCF in K562 cells using anti-HA antibody, scale bar = 5 μm. **D**, **E** Functional assays of CTCF mutants in K562 cells including: **D** MTT proliferation; and **E** colony forming assay in Methocult. Data represent the mean ± s.e.m for 3 experiments each performed in triplicate. Statistical analysis was performed using a one-way ANOVA with Tukey’s multiple comparisons test for pairwise comparisons between control, WT and mutant (ns = not significant; *p < 0.05; **p < 0.01; ****p < 0.0001)

To determine the functional consequences of CTCF ZF mutations, we examined missense mutations that had been detected in acute lymphoblastic leukaemia (ALL) samples: L309P (T-ALL; Mullighan unpublished), R339Q [39], R377H [44] and G420D (diagnosis and relapsed hyperdiploid B-ALL; Mullighan unpublished) (Fig. 2A, Supplementary Table 1). R377H occurs within an inter-ZF region, L309P affects the conserved intra-ZF Leu/Met residue, whilst G420D and R339Q both occur at key DNA-contacting residues + 2 and + 6, respectively (Fig. 2A). We included R339W as a positive control as it was first identified in Wilms’ tumour as a potential ‘change-of-function’ mutation that abrogated DNA binding to a subset of CTCF sites regulating genes involved in cell proliferation [36]. All five mutations exhibit high Polyphen scores, indicating they significantly impact CTCF function (Fig. 2A).

We introduced these mutations into HA epitope-tagged human CTCF within a lentiviral expression vector that co-expresses eGFP via a 2A peptide [35]. We transduced K562 erythroleukaemia cells with CTCF WT and mutant constructs and showed that ectopic CTCF expression occurred at similar levels and above endogenous CTCF levels (Fig. 2B). Immunofluorescent staining for ectopic HA-tagged CTCF indicated that all CTCF mutants maintained nuclear localisation similar to WT CTCF (Fig. 2C). We next examined cell growth and showed that WT CTCF overexpression suppressed cellular proliferation (*p* < 0.0001) consistent with it being a tumour suppressor and as previously shown [35] (Fig. 2D). Mutants L309P, R377H and R339W abrogated the tumour suppressive effect of CTCF and exhibited cellular proliferation similar to the empty vector control (all *p* < 0.0001 compared to WT), whilst R339Q had an intermediate effect on CTCF’s anti-proliferative function (*p* < 0.0001 compared to WT; *p* < 0.001 compared to control, Fig. 2D). K562 cells expressing CTCF G420D exhibited similar proliferation to WT CTCF (Fig. 2D). We next performed clonogenicity assays and showed that WT CTCF suppressed the colony-forming abilities of K562 cells as expected (*p* < 0.0001, Fig. 2E). Again, L309P, R377H and R339W abrogated the suppressive effect of CTCF on colony formation (*p* < 0.0001) whilst R339Q had an intermediate effect compared to both control (*p* < 0.0001) and WT (*p* = 0.011). Remarkably, G420D exhibited gain-of-function by further reducing the clonogenic capacity compared to WT (*p* = 0.0103, Fig. 2E).

### CTCF ZF mutations disrupt DNA binding and transcriptional regulation

**Fig. 3:**
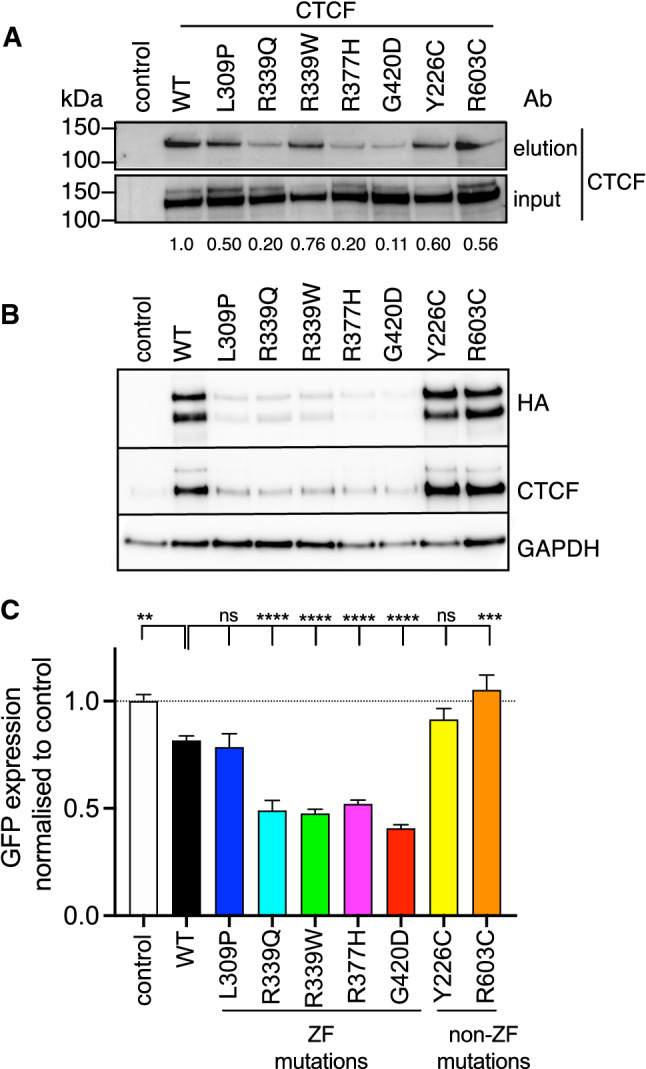
CTCF ZF mutants reduce DNA binding and transcriptional regulation. **A** CTCF DNA binding assay performed with in vitro transcribed and translated CTCF protein (WT and mutants) and a biotinylated dsDNA probe representing the core CTCF binding site. Eluted and input samples were probed for CTCF protein by Western blot; numbers at the bottom indicate band densitometric values after normalisation to input. **B**, **C** Control (eGFP alone), CTCF WT- or mutant-containing lentivector plasmids were transfected into HEK293T cells for 48 h. **B** Representative Western blots (of 3 replicates) indicating ectopic (HA-tagged) CTCF, total CTCF and GAPDH loading control after transfection of HEK293T cells. **C** GFP mean fluorescence intensity (MFI) detected after 48 h and normalised to eGFP empty vector control set as 1.0. Data represent the mean ± s.e.m for 3 experiments each performed in triplicate except for the Western blots which are only single replicates. Statistical analysis was performed using a one-way ANOVA with Tukey’s multiple comparisons test for pairwise comparisons between control, WT and mutant (ns = not significant; **p < 0.01; ***p < 0.001; ****p < 0.0001)

We next examined the impact of ZF mutations on DNA binding by CTCF. Frequently occurring N-and C-terminal somatic missense mutations (Y226C and R603C, respectively) were included as additional controls. Y226 is a key anchoring residue in the interaction of CTCF with the SA2-SCC1 cohesin complex [46], whilst R603 resides within an RNA binding region [47]. Using the core CTCF binding site [45] as a biotinylated double-stranded probe bound to streptavidin beads, we showed that in vitro transcribed and translated CTCF WT protein robustly bound to the core binding site (Fig. 3A). CTCF ZF mutants R339Q, R377H and G420D exhibited diminished DNA binding (Fig. 3A), whereas R339W and L309P displayed similar CTCF binding ability to non-ZF mutants (Fig. 3A). These results are consistent with a previous report [36] suggesting that CTCF ZF mutations disrupt DNA binding in a sequence-specific context.

To examine the impact of CTCF ZF mutation on transcriptional regulation, a lentiviral plasmid [35] encoding WT or mutant CTCF, but with no internal promoter (Supplementary Fig. 3A), was transfected into HEK293T cells followed by quantitation of CTCF protein and eGFP fluorescence levels. Strikingly, all CTCF ZF mutants exhibited decreased levels of ectopic CTCF expression to levels ~ 10–20% of WT, whilst non-ZF mutants demonstrated levels comparable to, or higher than, WT control (Fig. 3B, Supplementary Fig. 3B). Notably, this decrease was not due to mutant CTCF protein instability as sustained expression was stable (Fig. 2B). Furthermore, decreased eGFP expression was observed with CTCF mutants R339Q, R339W, R377H and G420D compared to WT (all *p* < 0.0001) (Fig. 3C). L309P and Y226C had no impact; however, non-ZF mutation R603C exhibited higher eGFP expression than WT (*p* = 0.0003), but similar to empty vector control (Fig. 3C). Importantly, over a dozen putative CTCF binding sites were predicted in the vector backbone including within the chimeric CMV-long terminal repeat (LTR) promoter that drives viral vector RNA expression (Supplementary Fig. 3A). As CTCF ZF mutants exhibited diminished CTCF protein expression and lower eGFP expression (Fig. 3B, C), these data indicate that CTCF ZF mutants may negatively impact on CTCF’s transcriptional regulator function. As further confirmation of this, we expressed the same eGFP- and HA-CTCF-containing cassette from a different lentiviral plasmid, but which now contained a strong internal (ubiquitin C) promoter (Supplementary Fig. 3C). We observed equivalent levels of WT and mutant protein (Supplementary Fig. 3E), however, CTCF ZF mutant transfections exhibited diminished eGFP expression compared to WT (Supplementary Fig. 3D), indicating CTCF’s transcription regulatory activity was negatively impacted.

**Fig. 4:**
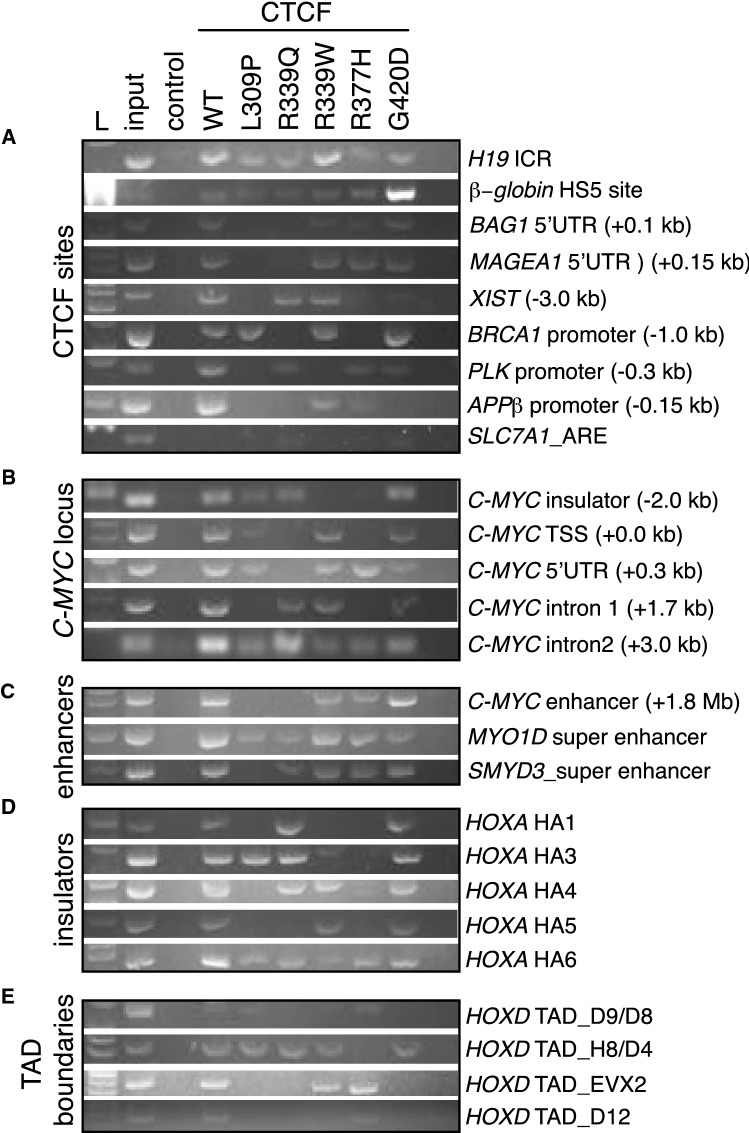
Differential DNA binding exhibited by ZF-mutant CTCF. ChIP-PCR of HA-tagged WT and mutant CTCF expressed in K562 cells; L = 100 bp ladder, input is total genomic DNA before ChIP, Control = eGFP empty vector. Diverse CTCF sites were examined: including **A** archetypal CTCF sites; **B** the *C-MYC* locus; **C** enhancers; **D** insulators; and **E** TAD boundaries. Where relevant, the genomic distance from the TSS is indicated in brackets. The *SLC7A1* androgen response element (ARE) was used a negative control for CTCF binding. See Supplementary Table 4 listing references for known CTCF sites and their chromosomal locations

To examine the impact of CTCF ZF mutation on DNA binding more globally, we performed chromatin immunoprecipitation (ChIP) to determine if ZF-mutant disruption of transcriptional regulation leads to abrogation or alteration of DNA binding at CTCF target sites. Notably, we achieved equivalent levels of HA-tagged WT and ZF mutant CTCF in K562 cells after lentiviral transduction (~ 15–20% for all, Supplementary Fig. 2). We then performed ChIP using an anti-HA antibody, followed by PCR amplification of known CTCF target sites (Fig. 4). We observed both WT and mutant CTCFs still associating with archetypal CTCF target sites such as the *H19* imprinting control region (ICR) and the β-globin hypersensitivity site HS5 (Fig. 4A). However, variegated CTCF mutant binding was detected at other cognate CTCF target sites proximal to the regulatory regions of *BAG1*, *MAGEA1*, *XIST*, *BRCA1*, *PLK* and *APPβ* (Fig. 4A). All CTCF ZF mutants exhibited a selective loss of DNA binding, with L309P, R339Q and R337H mutations exhibiting the greatest loss in binding (Fig. 4A–E). All CTCF mutants except G420D exhibited some loss of binding within the archetypal CTCF-regulated gene *C-MYC* (Fig. 4B). CTCF binding sites within known enhancers (Fig. 4C), insulator sites (Fig. 4D) and TAD boundaries (Fig. 4E) all showed selective binding by most CTCF ZF mutants. As CTCF binding is not completely abrogated at all sites, these data are consistent with CTCF ZF mutants displaying a change-of-function rather than loss-of-function.

### Molecular dynamics (MD) simulations explain CTCF loss- and gain-of-function ZF mutant phenotypes

**Fig. 5:**
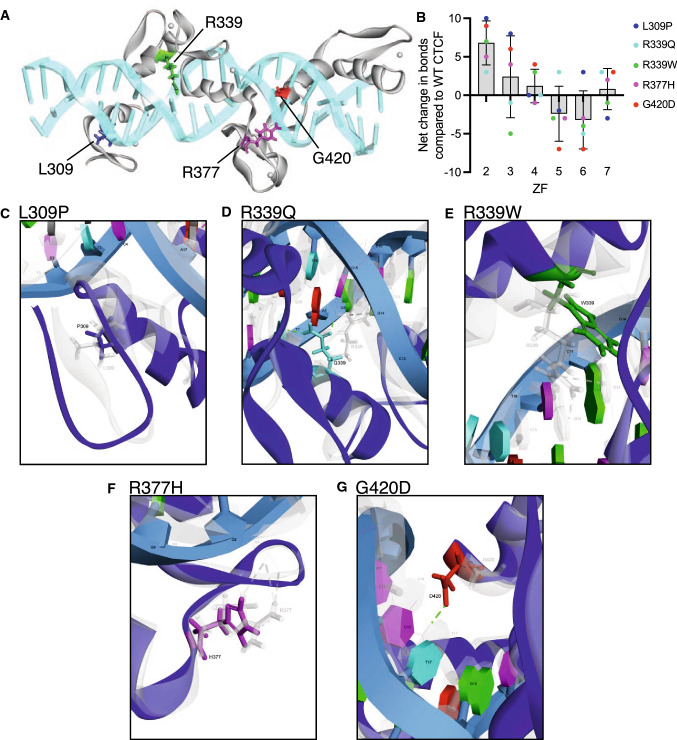
Homology modelling of CTCF ZF mutations. **A** CTCF ZF residues impacted by somatic mutation are depicted on the crystal structure model of ZFs 2–7 in association with DNA. Zinc molecules are shown as grey spheres. **B** Net change in bonds in individual CTCF ZFs following specific ZF mutations. Data shows the mean ± SD. **C**–**G** Overlay images of the normal (WT, grey) and mutant (blue) residues superimposed on the CTCF crystal structure: **C** L309P (L grey, P dark blue); **D** R339Q (R grey, Q cyan); **E** R339W (R grey, W green); **F** R377H (R grey, H magenta); **G** G420D (G grey, D red). Dashed lines indicate hydrogen bond pairing: old (grey) and new (green). DNA bases and their position relative to the 5′ end of the CTCF consensus are shown

To gain insights into the structural impact of these somatic mutations we modelled them on the published crystal structure of CTCF’s ZF domain (ZFs 2–7) in complex with DNA [45]. First, using molecular docking, the locations of the 4 mutated ZF residues were superimposed on the CTCF structure (Fig. 5A). Then, the folding free energy change (ΔΔ*G*) was individually calculated for all 5 resulting ZF mutations which indicated that each of the mutations were destabilising (Table 1). L309P is predicted to have the most severe impact on CTCF folding (ΔΔ*G* = 12.05 kcal/mol), compared to R339Q (ΔΔ*G* = 6.87 kcal/mol), R339W (ΔΔ*G* = 5.00 kcal/mol), R377H (ΔΔ*G* = 5.64 kcal/mol) and G420D (ΔΔ*G* = 1.91 kcal/mol). To investigate these mutations further, we used molecular dynamics (MD) simulations to model the impact of each ZF mutation on the whole structure by collating the total number of DNA bonds for each ZF before and after mutation (Table 1). All CTCF mutations examined perturbed local ZF-DNA bonds as well as bonds in adjacent and distal ZFs (Table 1). ZFs 4 and 7 which bind the most invariant nucleotides in the CTCF core consensus (Table 1) were the least affected by ZF domain mutations, irrespective of which ZF the mutation occurred in (Fig. 5B). Whereas, ZF6 was the most unstable ZF among the core DNA binding ZFs (Fig. 5B). Time evolution studies of secondary structure in WT and mutant CTCF ZF domains indicated that structural elements were stable at the location of each mutation (Supplementary Fig. 3). However, β-sheet-forming elements (red) were disrupted by: L309P (ZF2), R339Q, R339W (ZF3) and R377H (ZF4-5) between aa 353–363 in ZF4; and R339W, R377H and G420D (ZF6) between 295–305 in ZF2. Interestingly, in all mutants, the β-sheet and turn structure at aa 408–418 (ZF6) was also disrupted (Supplementary Fig. 3), consistent with our ZF-DNA bond analysis (Table 1, Fig. 5B).

**Table 1:** Consequences of CTCF ZF mutations on DNA binding and protein folding

		Number of ZF/DNA bonds predicted from molecular docking of WT and mutant CTCF ZF					
		WT	L309P	R339Q	R339W	R377H	G420D
	Core CTCF site		(ZF2)	(ZF3)	(ZF3)	(ZF4/5)	(ZF6)
ZF2		6	16	9	13	11	15
ZF3	A/GC/GT/C	9	17	8	4	13	15
ZF4	GGC	15	15	15	18	14	19
ZF5	GG/AN	24	22	27	21	21	17
ZF6	NNA	17	20	12	13	14	10
ZF7	NCA	14	11	17	13	16	17
Total		85	101	88	82	89	93
Net change			+ 16	+ 3	− 3	+ 4	+ 8
Mutation Energy (ΔΔ*G*, kcal/mol)			12.05	6.87	5.00	5.64	1.91
Effect			Destabilising	Destabilising	Destabilising	Destabilising	Destabilising

Using molecular dynamics simulations of WT and mutant CTCF, the number of bonds formed between CTCF ZF residues and the CTCF binding site was predicted for each ZF. The following bond categories were collated: hydrogen bond; electrostatic and hydrophobic. Using molecular docking, the effect of mutation on the CTCF ZF structure folding energy was measured by the change in minimum free energy (ΔΔ*G*) for WT and mutant ZF structures in the DNA-bound state. ΔΔ*G* values: > 0.5 kcal/mol are destabilising; − 0.5 to 0.5 kcal/mol are neutral; < − 0.5 kcal/mol are stabilising. The nucleotide triplet that each ZF binds in the 15 bp core CTCF site used for this model (5′-NCANNAGG/AGGCA/GC/GT/C-3′) [45] is shown. The ZF containing each respective mutation is shown in brackets

**Fig. 6:**
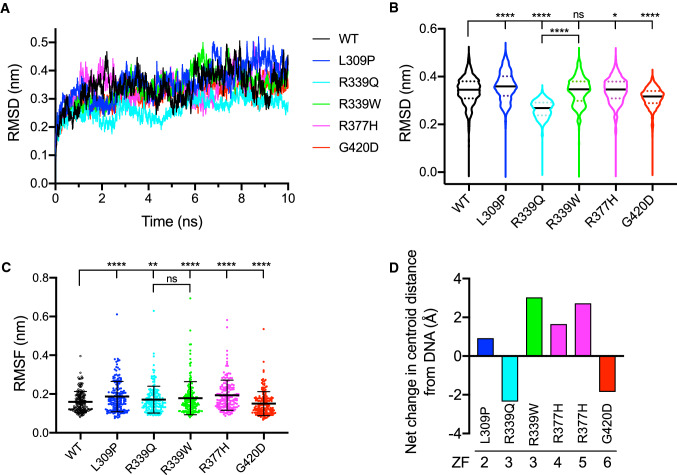
Impact of mutation on CTCF ZF domain conformational stability revealed by MD simulations. **A**, **B** Root-mean-square deviation (RMSD) measurements were calculated from the position differences of backbone atoms in the native (WT) and various mutant conformations. **A** Trajectories of all 5 mutants and WT CTCF are displayed over a 10 ns time span, measured at 2 ps intervals. **B** Violin plots of all RMSD measurements (0.000–10.000 ns, 5001 in total). In each plot, the solid black line indicates median and dashed coloured lines indicate quartiles. **C** Root-mean-square fluctuation (RMSF) measurements were obtained for all residues (*n* = 173) at each time point for the WT and mutant structures. In each plot, the mean ± SD is shown. In **B**, **C** the Wilcoxon matched-pairs signed rank test was applied to all paired measurements (ns = not significant; *p < 0.05; **p < 0.01; ****p < 0.0001). **D** The net change in distance (in Å) of the centre of mass (centroid) of the associated ZF domain from DNA compared to WT CTCF

To examine each mutation in more detail, we visualised the superimposed structures of WT and mutant CTCF ZF structures. L309 is oriented away from DNA and does not directly contact DNA either before or after mutation to Pro (Fig. 5C). Despite this, analysis of molecular interactions between neighbouring CTCF amino acid residues and DNA revealed 7 existing bonds were lost, whereas 12 new bonds were formed (Supplementary Table 3). Root-mean-square deviation (RMSD) measurements showed that the L309P mutation induced a substantial increase in the deviation of the ZF2 backbone compared to WT over the 10 ns simulation run (Fig. 6A,  B, *p* < 0.0001). Similarly, root-mean-square fluctuation (RMSF) measurements spanning the entire ZF 2–7 structure (Supplementary Fig. 4) indicated that there was a considerable increase in flexibility (*p* < 0.0001, Fig. 6C). Consequent to all the conformational changes, the distance of the ZF2 centroid from the DNA centroid was also increased (0.916 Å) in the L309P mutation (Fig. 6D).

Arginine 339 at DNA-binding position ‘ + 6’ within ZF3 directly contacted guanine (G14) and cytosine (C13) residues on one DNA strand via two hydrogen bonds and one cation-π bond, however, mutation to Q (R339Q) or W (R339W) abolished these bonds (Fig. 5D, E, Supplementary Table 3). Remarkably, Q339 formed two new hydrogen bonds: first, between the Gln side-chain carbonyl group and cytosine (C15); and second, between the side chain amide group and thymine (T7) on the complementary strand (Fig. 5D). Both mutations also disrupted the interaction of E336 with cytosine (C15), with 6 and 4 new bonds formed at neighbouring residues for Q339 and W339, respectively (Supplementary Table 3). MD simulations showed that the R339Q triggers less conformational deviation than WT or R339W (Fig. 6A, B, both *p* < 0.0001), however, over the entire ZF structure R339Q and R339W both exhibited more flexibility than WT (Fig. 6C, p = 0.0018 and *p* < 0.0001, respectively). Consequently, R339Q shifted ZF3 towards DNA (2.342 Å) and in the case of R339W, ZF3 moved away from DNA (3.021 Å) (Fig. 6D).

R377H, which occurs in an inter-ZF residue between ZF4 and ZF5, disrupted three hydrogen bonds that stabilise the interaction of R377 with the DNA phosphate moiety at guanine (G8) (Fig. 5F). Adding to this, 22 neighbouring molecular contacts are lost and 22 new bonds are formed (Supplementary Table 3). RMSD measurements show that R377H induced an increased deviation in the conformation over time (Figs. 6A, B, *p* = 0.0439) and increased flexibility in the entire ZF structure (Fig. 6C, p < 0.0001). As a result, both ZF4 and ZF5 were shifted away from the DNA phosphate backbone (1.643 Å and 2.718 Å, respectively, Fig. 6D).

Finally, CTCF modelling confirms glycine at position 420 and DNA-binding position ‘ + 2’ in ZF6 does not directly contact DNA (Fig. 5G). However, when mutated to aspartic acid (G420D), a new hydrogen bond is formed between the side chain carbonyl group and cytosine (C16) in the core consensus sequence (Fig. 5G). A net loss of 4 bonds at neighbouring residues was also observed (Supplementary Table 3). RMSD measurements showed that G420D exhibited decreased structural deviation during the simulation run (Figs. 6A, B, *p* < 0.0001) and decreased RMSF values compared to WT indicating reduced flexibility (*p* < 0.0001, Fig. 6C). Consequently, G420D resulted in ZF6 shifting 1.841 Å toward the DNA (Fig. 6D).

In summary, our data suggest that mutations R339W and R377H disrupted CTCF’s primary interactions with DNA and, along with the highly destabilising L309P mutation, are responsible for shifting ZF domains away from DNA. Importantly, R339Q and G420D both formed new primary bonds and the associated ZF domain moved nearer to the DNA.

## Discussion

Tumour-specific mutations in CTCF were described almost two decades ago [36]. In that seminal report, functional testing of some ZF mutations comprised electrophoretic mobility shift assays (EMSA) and a reporter assay. The key finding from these preliminary functional studies was that these somatic CTCF ZF mutations exhibited selective disruption in DNA binding to some, but not all, CTCF target sites, giving rise to the concept of ‘change-of-function’ mutations [36]. Since then the functional characterisation of CTCF ZF mutations has been limited, despite many landmark cancer genome studies reporting hundreds of missense somatic CTCF ZF mutations.

The potential genome-wide impacts of CTCF ZF mutation on DNA binding were examined through mutation in all 11 ZFs of a key Zn^2+^ co-ordinating histidine residue within the conserved C2H2 tetrahedron arrangement that co-ordinates ion binding [16]. This approach, whilst not directly emulating tumour-specific missense mutations, revealed that in all cases DNA binding was not completely abolished. Indeed, residual DNA binding ranged from ~ 15–80% depending on the position of the ZF [16]. Our previous report showed that the 3 CTCF ZF mutations most frequently occurring in endometrial cancers (K365T, R377H, P378L) had differing impacts on CTCF function when overexpressed [38]. The two inter-ZF mutations (R377H and P378L) abrogated CTCF’s anti-proliferative and anti-clonogenic effect in Ishikawa endometrial cancer cells. However, mutation of K365, a key DNA-binding residue at position ‘ + 3’ in ZF4, to threonine, had no impact on proliferation or colony formation [38], despite causing a 20-fold loss of CTCF DNA-binding affinity [45]. Interestingly, CTCF K365T conferred significantly increased resistance to UV-induced apoptosis in Ishikawa cells compared to WT CTCF, suggesting the first pro-tumourigenic role of CTCF ZF mutations [38].

Initial deletion mutagenesis studies of the CTCF ZF domain indicated ZFs 3–11 were required to bind the human *c-myc* promoter [48]. Further refinement was achieved in two studies, in which similar binding modes were confirmed on the chicken *c-myc* promoter requiring central ZFs 2–7 [48] or ZFs 3–8 [49]. Furthermore, the central ZFs 5–7 of CTCF were required for *APPβ* promoter binding, however, it was the peripheral ZFs which provided the stability in DNA binding [49]. Whilst gel mobility shift analysis confirmed that CTCF ZFs 4–7 were necessary and sufficient to bind to a core 12 bp consensus sequence [50], the first crystal structure of the CTCF ZF domain resolved that ZFs 3–7 bound the 15 bp core DNA consensus sequence [45]. Nakahashi et al. proposed a ‘saddle’ model containing a core CTCF motif (*C*) bound by central ZFs 4–7 as well as upstream (*U*) and downstream (*D*) modules bound by peripheral ZFs [16]. Yin et al. further refined this model by describing 4 CTCF binding site modules [51]. Modules 4, 3 and 2, spanning the 15 bp core CTCF consensus as well as downstream sequences, are bound by ZF3, ZFs 4–6 and ZF7, respectively; whilst upstream module 1 is bound by ZFs 9–11 [51]. These studies provide insights as to why mutations occurring in different CTCF ZFs may produce diverse effects on DNA binding and functional outcomes depending on where in the modular binding mode the mutant ZF residue occurs.

The five different somatic missense ZF mutations we examined in this study each occur in key positions within the central zinc fingers. Each mutation provided critical insights into CTCF structure–function relationships. The spatial arrangement of residues within the C2H2 ZF finger motif, including the flexible inter-ZF spacer, are critical to maintaining ZF structure, and are therefore very highly conserved [52]. Somatic ZF mutations did not affect overall CTCF protein stability or localisation within the nucleus when stably expressed. However, when transiently overexpressed, CTCF ZF mutants clearly decreased transcriptional activation compared to WT CTCF. Interestingly, our previous endometrial cancer study indicated that missense ZF-containing *CTCF* alleles were expressed at a higher frequency than WT alleles, when comparing RNA sequencing to DNA sequencing [38]. This suggested that the expression of aberrant CTCF was up-regulated to functionally compensate for a deficit in CTCF function. In our study, we observed differing impacts on CTCF-mediated cellular proliferation. These impacts on CTCF function are attributable to a change or gain in DNA-binding specificity.

L309 typifies an invariant hydrophobic residue (always Leu or Met) in the alpha-helical region of all CTCF C2H2 ZF fingers. Residues near the C-terminal end of each C2H2 ZF fold into an alpha helix, positioning key amino acids within the helix to interact directly with DNA [52]. L309 mutation to Pro (L309P) affected the thermodynamic stability of ZF2, most likely through the α-helical-breaking tendency of proline in water-soluble proteins. We confirmed increased RMSD and RMSF values during molecular dynamics simulations and a shift of CTCF away from DNA. Not surprisingly, despite some DNA-binding still being maintained, L309P exhibited loss-of-function in in vitro cell growth assays. R339 mutation to Q or W differentially impacted CTCF growth and colony-forming function and this too was explained by molecular dynamics simulations. For R339Q, the ZF domain of the CTCF shifts closer to DNA and two new hydrogen bonds are formed at Q339, explaining the intermediate loss-of-function cell growth phenotype observed with R339Q. R339W, which exhibits loss-of-function phenotypes, disrupts all primary DNA contacts and deflects ZF3 away from DNA. This is despite R339W still maintaining nearly half (47%) of DNA binding genome-wide [16].

R377 resides in one of the inter-ZF regions which act as bridges between ZFs to allow flexibility in the DNA-free form but stability in the DNA-bound form [45]. Our modelling showing that R377 contacts the DNA phosphate backbone was also confirmed by structural studies of the CTCF ZF domain bound to the *Pcdh* enhancer [51]. Hence, not all amino acids at canonical DNA binding positions in ZFs directly contact DNA, such that intra- and inter-ZF residues are also involved in DNA contacts [51]. The R377H mutation, which eliminates this DNA interaction, also destabilises neighbouring molecular interactions. R377H exhibited loss-of-function cell growth phenotypes in K562 erythroleukaemia cells, similar to our previous observations in endometrial cancer cells [38]. Remarkably, despite G420D exhibiting some loss of binding to target sites and loss of gene regulatory activity, a gain-of-function was observed as it suppressed colony formation to a greater extent than WT CTCF. Consistent with these phenotypes, G420D formed a new bond with DNA and resulted in ZF6 shifting towards DNA. Overall, these studies reveal that the location of the ZF missense mutation determines the impact it has on loss-, change- or gain-of-function in CTCF. We examined mutations in DNA-contacting residues, in a residue co-ordinating ZF folding, in the inter-ZF region and within or outside the central core consensus binding ZFs 4–7. Furthermore, the mutant amino acid residue side chain can also have a significant impact on cellular phenotypes.

Different ZF modules with identical DNA specificity residues (at positions − 1, + 2, + 3 and + 6) can bind different sequences, influenced by DNA sequence context and inter-ZF residue-residue interactions [53]. Furthermore, neighbouring ZFs may affect the DNA-binding conformation and specificity of a particular ZF [54]. Our data has revealed that those residues in close proximity to the mutant residue can lose existing bonds or acquire new DNA interactions. Similarly, missense ZF mutations in CTCF can destabilise the DNA-bound conformation of adjacent and distal ZFs. Thus, MD simulations have illuminated the broad and diverse impact that CTCF ZF mutations exert on DNA binding.

What remains to be determined is the impact somatic ZF missense mutations will have on rewiring genomic architecture. Topologically associating domains (TADs) are discrete territories, compartmentalising the genome into independent, often evolutionarily conserved domains [22, 23, 25, 55, 56]. TADs are characterised by frequent CTCF-mediated contacts within domains and a low frequency of contacts between domains [57]. These TADs are themselves demarcated into sub-megabase sub-TADs and loop domains, often differentially co-ordinated by CTCF interaction with other architectural proteins such as cohesin [21, 26, 58]. Deletion or inversion of CTCF sites at TAD boundaries can drastically affect gene regulation, leading to ectopic activation of gene expression due to illegitimate promoter and enhancer interactions, often with pathogenic consequences [28, 29, 59, 60]. In cancer, genetic alteration or hypermethylation of CTCF sites at TAD boundaries can disrupt chromatin topology and lead to aberrant activation of oncogenes [61–63]. The global impact of somatic missense mutations in CTCF, which typically only occur on one allele and cause *CTCF* haploinsufficiency, remains to be investigated.

## Conclusions

Over the last decade, unprecedented insights into CTCF’s essential role in genome organisation and architecture have been revealed via the generation of high-resolution maps of chromatin interactions by chromosome conformation capture (3C)-based techniques. However, the structure–function relationships of CTCF mutations, particularly within the DNA-binding ZF domain, have not been investigated. We reveal that the CTCF ZF domain is significantly mutated in cancer, with ZF-specific missense mutations impacting CTCF’s anti-proliferative capacity, DNA-binding and gene regulatory activities. Strikingly, we observed a broad spectrum of functional impacts ranging from complete, partial or no loss-of-function in cellular growth phenotypes and transcriptional regulation, as well as gain-of-function, resulting from the formation of new bonds between the mutant ZF and DNA. Our MD simulations revealed that all CTCF ZF mutations were destabilising, with the loss or gain in DNA binding not just localised to the mutant residue. This highlights the importance of understanding structure–function relationships in normal and mutated CTCF. As *CTCF* exhibits haploinsufficiency in cancer, the interplay between mutant and wildtype CTCF at specific loci and at target sites genome-wide remains an unanswered question. Understanding how somatic CTCF ZF mutations affect chromatin topology globally will be the next frontier in understanding the molecular pathophysiology of cancer.

## Materials and methods

### Cell lines

Human erythroid leukaemia (K562) cells (source: ATCC CCL-243) were grown in RPM1 1640 medium and human embryonic kidney (HEK293T) cells (source: D. Baltimore, California Institute of Technology) were cultured in DMEM. All basal media were supplemented with 10% FCS (v/v), penicillin (100 U/mL) and streptomycin (100 μg/mL). All human cell lines have been authenticated by short tandem repeat profiling (Cellbank, Australia).

### Reagents: expression vectors and antibodies

The lentiviral vector pCCLteteGFP2AHAhCTCF [35] was used to express CTCF ZF mutations. PCR amplicons containing ZF mutations (L309P, R377H, G420D) were generated by splice overlap extension PCR and were cloned in using *BmgBI/ClaI*. R339Q and R339W mutations were created by gene synthesis from DNA2.0 and sub-cloned using *Bsu36I/Tth111I*. Y226C and R603C were synthesised as Geneblocks (IDT) and cloned into *BstX1*/*BstXI* sites and *PstI/BlpI* sites, respectively. In addition, all CTCF mutations were sub-cloned into an additional pFUW-based lentiviral plasmid backbone (pFUW-eGFP-2A-HAhCTCF). For in vitro transcription/translation experiments pcDNA3.1-FLAG-CTCF mutants were made using Gibson assembly of PCR-amplified CTCF mutants. Primary antibodies include: CTCF rabbit monoclonal (#3418, Cell Signaling Technology; 1:5000), HA epitope mouse monoclonal (HA.11, Covance; 1:5000) and GAPDH (ab8245, Abcam; 1:5000). Secondary antibodies include: rabbit or mouse antibodies conjugated to horseradish peroxidase (HRP, Millipore; 1:5000).

### Retroviral and lentiviral transduction

Viral supernatants were produced by calcium phosphate transfection of HEK293T cells: with pJK3, pCMVTat and pL-VSV-G packaging plasmids used to produce replication-defective retroviruses; and pRSV-Rev, pMDLg/p.rre and pMD2.VSV-G used to produce replication-defective lentiviruses. Viral supernatants collected after 24–48 h were 0.45 μM-filtered and snap-frozen or concentrated by ultracentrifugation for 2 h at 20,000 rpm in a SW28 Beckman rotor. Viral supernatant was resuspended on ice in 10% (v/v) FCS/DMEM at 1/100th of the original volume. Attached cells (1–5 × 10^5^/well) were seeded in 6-well plates before the addition of fresh medium containing viral supernatant (~ 5 × 10^5^ transducing units) and Polybrene (8 μg/mL; Sigma) and ‘spin-oculated’ for 90 min at 1500 rpm. The supernatant was replaced with medium 12 h post-transduction and fluorescent cells were purified 24 h later by fluorescence-activated cell sorting (FACS; > 95% purity on re-analysis) using a FACS Influx (Becton Dickinson, BD). K562 cells (~ 5 × 10^5^/mL) in 1 mL medium with 4 μg/mL Polybrene were placed in a 5 mL capped FACS tube and transduced with viral supernatant for 90 min by ‘spin-oculation’. The cells were resuspended, incubated at 37 °C for 4 h before removal of viral supernatant. For in vitro assays, cells were either plated out immediately or allowed to recover after sorting for 48–72 h in medium containing 100 μg/mL Normocin (Invivogen).

### Immunofluorescence

Transduced K562 cells (1 × 10^6^) were fixed with an equal volume of 4% (w/v) formaldehyde for 20 min at room temperature (RT). Cells were centrifuged at 2000 rpm for 3 min and resuspended in PBS twice. Cells (5.0 × 10^5^) were allowed to settle onto coverslips coated with poly-d-lysine, before drying and permeabilisation with Triton X-100 0.5% (v/v) in PBS for 10 min at RT. Cells were rinsed three times in PBS and blocked in 3% (w/v) BSA/PBS for 40 min at RT. Cells were rinsed three times in PBS and incubated with mouse anti-HA antibody (1:500, HA.11, Covance) for 90 min at RT. Cells were rinsed three times in PBS and incubated with F(Ab’)2-goat anti-mouse IgG-Alexa 594 (1:500, #A11020, ThermoFisher Scientific) and DAPI (1:10,000, #D1306, Life Technologies) for 40 min at RT. Cells were rinsed three times in PBS and mounted using Prolong Gold Antifade (Life Technologies). Slides were imaged at 60 × using the DeltaVision Personal (Applied Precision) and the DAPI, FITC and A594 filters. Images were analysed after deconvolution using Volocity software.

### Western blot analysis

Protein extracts were prepared with cell lysis buffer containing 20 mM TrisCl (pH 8), NaCl (150 mM), 1% (v/v) Triton X-100, 0.1% (v/v) SDS, 0.5% (w/v) sodium deoxycholate and EDTA-free protease inhibitor cocktail (cOmplete, Roche Life Science), prior to separation using denaturing sodium dodecyl sulfate–polyacrylamide gel electrophoresis **(**SDS-PAGE). Proteins were transferred in a semi-dry transfer apparatus to PVDF membrane before immunoblotting. Membranes were blocked in PBST containing 20% (v/v) BlokHen (AvesLab) or PBST containing 0.3% (w/v) BSA, 1% (w/v) polyvinylpyrrolidone, 1% PEG (mw 3350). Protein expression was detected using primary antibodies followed by washing and staining with appropriate secondary antibodies against rabbit, goat or mouse IgG conjugated to horseradish peroxidase (HRP). The HRP substrate SuperSignal® Chemiluminescent Substrate (Pierce) was detected on a Kodak Imagestation 4000R Pro or BioRad Chemidoc Touch. Blots were stripped with ReBlot Plus (Merck Millipore) prior to re-probing with protein loading control antibodies.

### Mutation and Bioinformatic analysis

CTCF mutations were obtained from the Catalogue of Somatic Mutations in Cancer (COSMIC) portal, The Cancer Genome Atlas (TCGA) cBIO portal and published reports (see Supplementary Table 1). Single nucleotide variants were obtained from dbSNP. The potential impact of mutations was determined using Polyphen-2. All amino acid alignments were performed using the Clustalw algorithm within MacVector. A raw alignment of CTCF ZFs was exported into Weblogo (weblogo.berkeley.edu/logo.cgi) to generate a sequence logo. The maximum sequence conservation for an amino acid is log_2_20 ~ 4.32 bits. CTCF target sites in CTCF-expressing plasmid pCCLteteGFP2AHA-hCTCF were predicted using MatInspector (Genomatix).

### Cell biological assays

Cell proliferation was assessed by 3-(4,5-methylthiazol-2-yl)-2,5-diphenyltetrazolium bromide (MTT) assay (Merck Millipore). K562 cells (5000/well) were plated in triplicate in a 96-well plate and proliferation was assessed over 4 days by the addition of MTT at 37 °C overnight. The reaction was quenched with isopropanol/HCl and then absorbance was measured at 572 nm using a Wallac 1420 Victor plate reader (Perkin Elmer). The clonogenic capacity of K562 cells was measured by plating 5000 cells diluted in Iscove’s Modified Dulbecco Medium (Life Technologies) containing 3 mL Methocult GF H4230 (Stem Cell Technologies) and plated in triplicate in 35 mm gridded plates and incubating for 8–10 days.

### Chromatin immunoprecipitation

K562 cells (1 × 10^6^ in 1 mL) were transduced with 10–60 uL viral supernatant of the control (eGFP only), CTCF WT and five CTCF mutants. After 72 h the cells were assessed by flow cytometry (LSR Fortessa, Becton Dickinson) and shown to vary between ~ 14–21% expression. For each chromatin immunoprecipitation (ChIP), 5 × 10^6^ cells were cross-linked with 1% (w/v) formaldehyde for 10 min and then quenched with 1 M glycine to a final concentration of 20 mM. Nuclear lysates were sonicated for 25 cycles, 30 s on, 30 s off using a Bioruptor sonicator (Diagenode). For each immunoprecipitation, 3 μg of rabbit polyclonal antibody against the HA epitope (ab9110, Abcam) was used. Magna ChIP™ Protein A/G conjugated magnetic beads (Millipore) were used to immunoprecipitate antibody-bound chromatin complexes, and all subsequent steps were performed according to the manufacturers’ instructions. After de-crosslinking, phenol/chloroform extraction, and ethanol precipitation, PCR was performed on genomic DNA targets using Phusion polymerase with GC buffer (Finnzyme). PCR primers spanning experimentally validated CTCF targets sites were designed from previous reports (see Supplementary Table 4). A more detailed protocol is available on request.

### Transfection of WT and mutant CTCF

HEK293T cells (1 × 10^5^) were plated into 12-well plates, 18 h before transfection. In each transfection, pCCLteteGFP2AHACTCF WT, mutant or empty vector (0.5 μg) was combined with 2 μL Lipofectamine 2000 (ThermoFisher) in OptiMEM medium (Gibco) according to the manufacturers’ instructions. After 48 h, cells were detached and assessed for eGFP by flow cytometer (LSR Fortessa, Becton Dickinson) and then lysed with cell lysis buffer.

### DNA binding assay

Forward and reverse oligonucleotides (100 μM each) representing the core CTCF binding site used in CTCF ZF X-ray crystallography studies [45] (5′AGGACCAGCAGGGGGCGCA-3′ and 5′biotin-TGCGCCCCCTGCTGGTCCT-3′, respectively) were used to generate a probe for DNA binding assays. Both oligos were annealed at a 1.5:1.0 molar ratio in 2 × annealing buffer (100 mM NaCl, 20 mM Tris–HCl pH 8.0, 2 mM EDTA) in a thermocycler (94 ^o^C 10 min, decreasing 1 ^o^C per min and held at 4 ^o^C). This generated a double-stranded oligonucleotide probe (20 μM) in a total volume of 60 μL. The probe was combined with 340 μL of DNA binding buffer (DBB) (1 M NaCl, 10 mM Tris pH 8.0, 1 mM EDTA, 0.05% (v/v) IGEPAL) and then immobilised onto streptavidin-conjugated Sepharose beads (Cytiva 17-5113-01, Sigma) pre-washed 3 × with ice-cold TBS (50 mM Tris–HCl pH 7.5, 150 mM NaCl), and incubated for 3 h at 4 °C with rotation. The bead/DNA mixture was washed 4 × with DBB followed by 4 × washes with Protein Incubation buffer (PIB) (250 mM NaCl, 50 mM Tris pH 8.0, 0.25% (v/v) IGEPAL, 1 × EDTA-free Protease Inhibitor Cocktail (cOmplete, Merck)) before adding it to recombinant CTCF protein, which was synthesised as described below.

Recombinant CTCF proteins were synthesised using the TnT® Quick Coupled Transcription/Translation System kit (#L1170, Promega) according to the manufacturer’s protocol. Plasmid DNA (3 μg) was mixed with the provided reagents (TNT® T7 Quick Master Mix, Methionine, 1 mM) plus RNAse-OUT (1 μL) and incubated for 3 h at 30° C. Following incubation, input (50 μL) was set aside before combining the recombinant protein mixture with beads/DNA and rotating for 3 h at 4 °C. The solution was washed 4 × with PIB to wash away unbound protein and CTCF protein was eluted in 40 μL 1 × LDS reagent (Thermo Fisher). Input and eluate samples were run on Western blots and probed with anti-CTCF antibody.

### Structural modelling and MD simulations

A 3.2 Å X-ray diffraction crystal structure representing the CTCF ZFs 2–7 / DNA complex (PDB: 5T0U) [43] was used as the initial template to prepare CTCF mutant models. The template was optimised using ‘Prepare Protein’ and ‘Energy Minimisation’ protocols available in Biovia Discovery Studio (DS) 2017R2 software suite. Initial mutant models (L309P, R339Q, R339W, R377H and G420D) were built using ‘Build and Edit Protein’ tool in DS by substituting original amino acid residues for the respective mutant. These mutant models were further optimised for their minimum energy confirmation using the steepest descent algorithm in DS with a non-bonded lower cut-off distance of 10 Å. Impact of mutations on protein stability was analyzed using ‘Calculate Mutation Energy (Stability)’ protocol in DS. The protocol calculates the difference in the free energy of folding (ΔΔ*G*_mut_) between the mutant structure and the wild type protein as follows:

$$\Delta \Delta G_{{{\text{mut}}}} \, = \,\Delta \Delta {\text{Gfold}}_{{({\text{mutant}})}} {-\!\!-}\Delta \Delta {\text{Gfold}}_{{({\text{wildtype}})}}$$

where ΔΔGfold is defined as the free energy difference between the folded and unfolded state of the protein. The unfolded state is modelled as a relaxed protein in extended conformation with the mutated residue in the centre.

To further analyse the impact of the mutation on CTCF binding ability to DNA, we first superimposed mutant CTCF models onto the wildtype CTCF model in complex with DNA using Chimera version 1.14. Furthermore, we performed MD simulations of WT and all the mutant models using GROMACS version 4.5.3. The system (CTCF mutant model in complex with DNA) was placed in the centre of a cubic box with at least 1 nm from the box edges. The ions (Na^+^ and Cl^−^) were added to the system for neutralising and preserving at a physiological concentration (0.15 M). The protocol consisted of successive rounds of energy minimisation, annealing, equilibration, and trajectory production in implicit solvent represented by the generalised Born/solvent-accessible surface area model and using a distance cut-off of 10 Å to short-range, non-bonded interactions. Keeping backbone atoms restrained, the protein was relaxed with 50,000 steps of energy minimisation, followed by annealing with a 60–300 K temperature ramp applied over 100 ps. In equilibration, the temperature was maintained at 300 K using Langevin dynamics for 50,000 steps over 100 ps. Production simulations were performed in the isothermal-isobaric ensemble, keeping both the DNA fragment and CTCF unconstrained. Bonds between hydrogen and heavy atoms were constrained at their equilibrium length using LINCS algorithm. Root-mean-square deviation (RMSD), root-mean-square fluctuation (RMSF), secondary structure and interaction energy analyses were carried out using GROMACS for the entire simulation run. All non-bonded interactions for the final poses of CTCF wildtype and mutants in complex with DNA were identified using DS.

## Supplementary Information

Below is the link to the electronic supplementary material.

Supplementary Figure 1 Predicted phenotypes of missense CTCF SNPs and somatic mutations. (A) The mean Polyphen score of missense SNPs and somatic mutations in CTCF is shown. (B) Proportion of the transition and transversion mutations for SNPs and somatic missense mutations in CTCF; ratio in brackets. Data represent the mean ± SD with statistical analysis performed using Mann-Whitney U-test (A) or Chi-square test (B) (****, p<0.0001) (XLS 203 KB)

Supplementary Figure 2 WT and mutant CTCF expression in K562 cells. Flow cytometry of eGFP expression achieved from transduction of HA-tagged WT and mutant CTCF lentiviral vectors in K562 cells. Cells were lysed for immunoblot (Figure 2B), prepared for immunofluorescence (Figure 2C) and subjected to formaldehyde cross-linking for ChIP (Figure 4). (PDF 73 KB)

Supplementary Figure 3 Validation of the negative impact of CTCF mutation on gene regulation. A) Schematic of the pCCLteteGFP-2A-HAhCTCF lentivector plasmid used to measure CTCF gene regulatory activity. The schematic highlights the plasmid map with cis-regulatory elements including the chimeric CMV-5’LTR driving viral vector RNA transcription. The eGFP-2A-HAhCTCF cassette is not linked to any internal promoter but is regulated by doxycycline ‘dox-off’’ when integrated into the genome. The expected protein products are also displayed. Predicted CTCF binding sites on both sense and antisense strands are represented by horizontal lines, respectively. B) Densitometry of ectopic and endogenous CTCF expression normalised to GAPDH, shown relative to WT CTCF set as 1.0. Data represents 3 independent experiments. C) Schematic of the pFUW-eGFP-2A-HAhCTCF lentivector plasmid used to independently confirm CTCF gene regulatory activity. The eGFP-2A-HAhCTCF cassette is driven by a strong internal Ubiquitin C (UbC) promoter. D) GFP mean fluorescence intensity (MFI) detected after 48 h and normalised to WT CTCF set as 1.0. Data represent the mean ± s.e.m for 4-5 experiments each performed in triplicate. E) Representative Western blots of pFUW-eGFP-2A-HA-hCTCF WT and mutant constructs transfected into HEK293T cells. Statistical analysis for B) & D) was performed using a one-way ANOVA with Tukey’s multiple comparisons test for pairwise comparisons between control, WT and mutant; *, p<0.05; **, p<0.01; ***, p<0.001, ****, p<0.0001). (XLSX 20 KB)

Supplementary Figure 4 Time evolution of secondary structure in WT and mutant CTCF ZF structures. The Dictionary of Secondary Structure of Proteins (DSSP) classification of secondary structure was calculated for all amino acid residues over the 10 ns time course: (A) WT; (B) L309P; (C) R339Q; (D) R339W; (E) R377H; and (F) G420D. Arrows indicate the position of each mutation. (XLSX 16 KB)

Supplementary Figure 5 Flexibility of CTCF ZFs before and after mutation measured by MD simulations. (A-D) Backbone RMSF values during the MD simulations for mutant CTCF ZFs compared to WT, spanning their associated ZF domain; dotted vertical line indicates position of mutation. (A) L309P; ZF2 (B) R339Q & R339W; ZF3 (C) R377H; ZF4 & 5, (D) G420D; ZF6. (PDF 5317 KB)

## Data Availability

All data generated or analysed during this study are included in this published article (and its supplementary information files). Additionally, PDB files for CTCF ZF mutant conformations are available from ModelArchive, accession IDs: L309P, ma-58jmu; R339Q, ma-qxtk8; R339W, ma-1xibx; R377H, ma-0uley; G420D, ma-uaxe1.

## Abbreviations

ChIP: Chromatin immunoprecipitation
CTCF: CCCTC-binding factor
DMEM: Dulbecco’s Modified Eagle Medium
EMSA: Electrophoretic mobility shift assay
FACS: Fluorescence-activated cell sorting
HA: Haemagglutinin
ICR: Imprinting control region
MD: Molecular dynamics
MTT: 3-(4,5–^1,2^Methylthiazol-2-yl)-2,5-diphenyltetrazolium bromide
PBST: Phosphate buffered saline Tween 20
PDB: Protein Data Bank
RMSD: Root-mean-square deviation
RMSF: Root-mean-square fluctuation
RPMI: Roswell Park Memorial Institute
SNP: Single nucleotide polymorphism
TAD: Topologically associating domains
TF: Transcription factor
WT: Wildtype
ZF: Zinc finger
