# Supplementary material for: Structure–function relationships explain CTCF zinc finger mutation phenotypes in cancer
Source: Cell Mol Life Sci. 2021 Oct 16;78(23):7519–36. doi: 10.1007/s00018-021-03946-z (PMC8629902; doi:10.1007/s00018-021-03946-z)
Supplement: Supplementary file 2 — Supplementary Figure 2 WT and mutant CTCF expression in K562 cells. Flow cytometry of eGFP expression achieved from transduction of HA-tagged WT and mutant CTCF lentiviral vectors in K562 cells. Cells were lysed for immunoblot (Figure 2B), prepared for immunofluorescence (Figure 2C) and subjected to formaldehyde cross-linking for ChIP (Figure 4). (PDF 73 KB) [file 18_2021_3946_MOESM2_ESM.pdf]

Supplementary Table 2 Analysis of the distribution of missense somatic mutations and SNPs in CTCF. The number of expected mutations was determined from the proportion of mutations expected if they were evenly distributed between each domain. The observed/expected (O/E) ratio confirms if there is a de-enrichment (<1.0) or an enrichment (>1.0) of non-synonymous changes. Statistically significant differences are indicated in bold calculated using the Chi-square test.

CTCF somatic missense mutations

Domain	*Observed*	*Expected*	*Observed/Expected Ratio*	*P*
N	94	150	*0.63*	***P*<0.0001**
ZF	266	181	*1.47*	***P*<0.0001**
C	54	83	*0.65*	***P*=0.0067**
total	414	414		

CTCF SNPs

Domain	*Observed*	*Expected*	*Observed/Expected Ratio*	*P*
N	94	74	*1.27*	***P*=0.0399**
ZF	42	88	*0.48*	***P*<0.0001**
C	67	41	*1.63*	***P*=0.0032**
total	203	203		
