# Supplementary figures and images for: Structure–function relationships explain CTCF zinc finger mutation phenotypes in cancer
Source: Cell Mol Life Sci. 2021 Oct 16;78(23):7519–36. doi: 10.1007/s00018-021-03946-z (PMC8629902; doi:10.1007/s00018-021-03946-z)

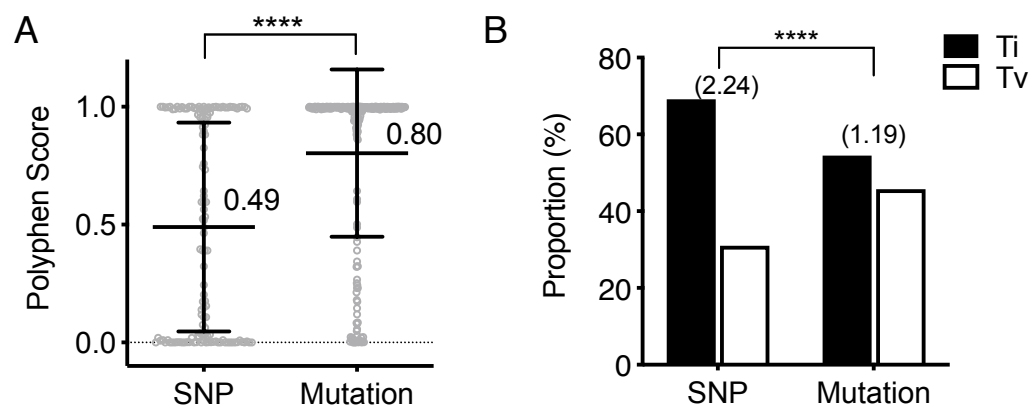

Bailey *et al* Supplementary Figure 2

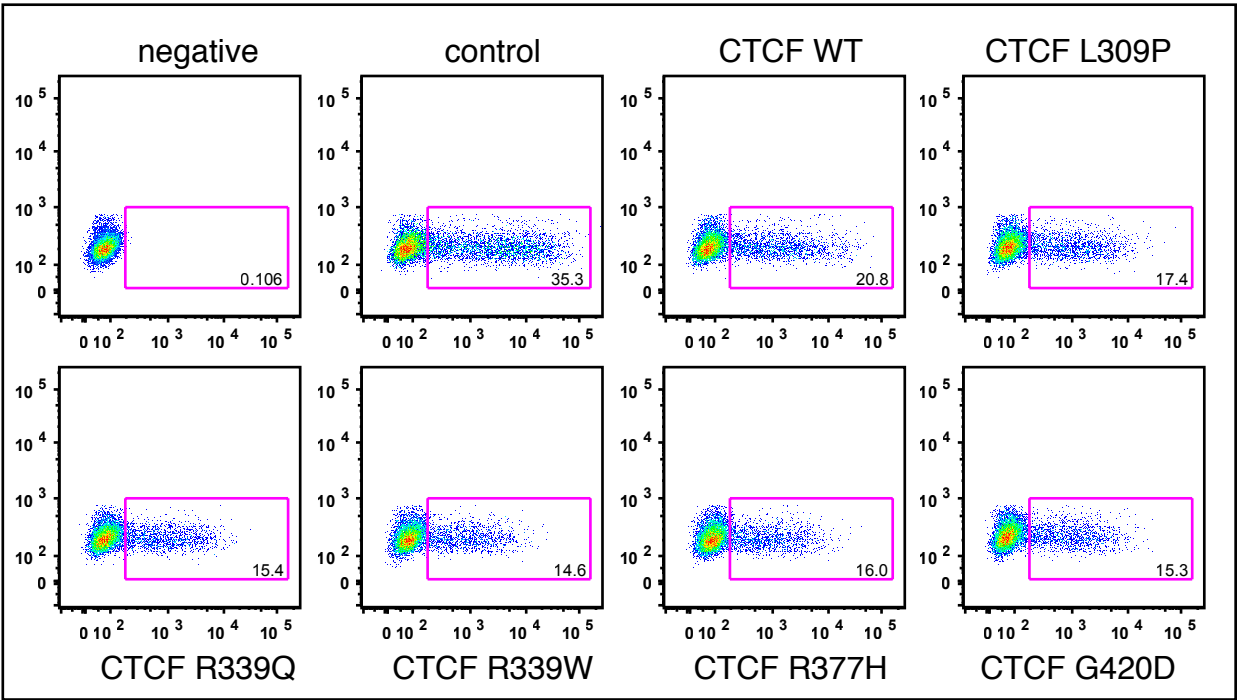

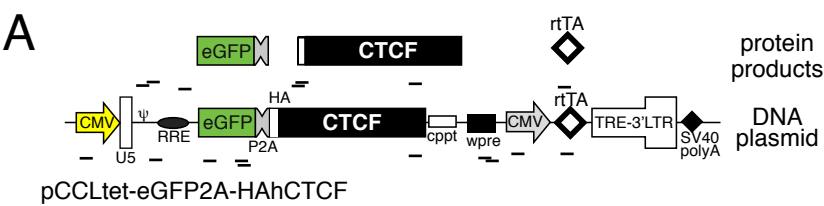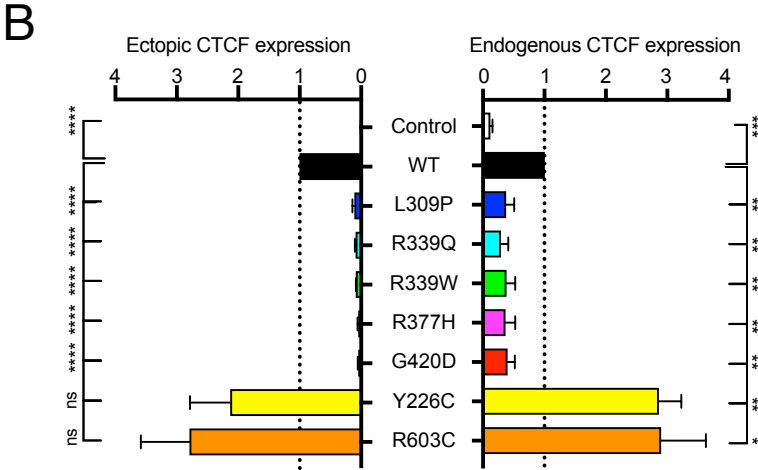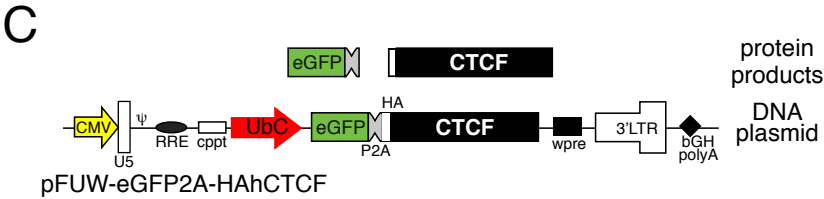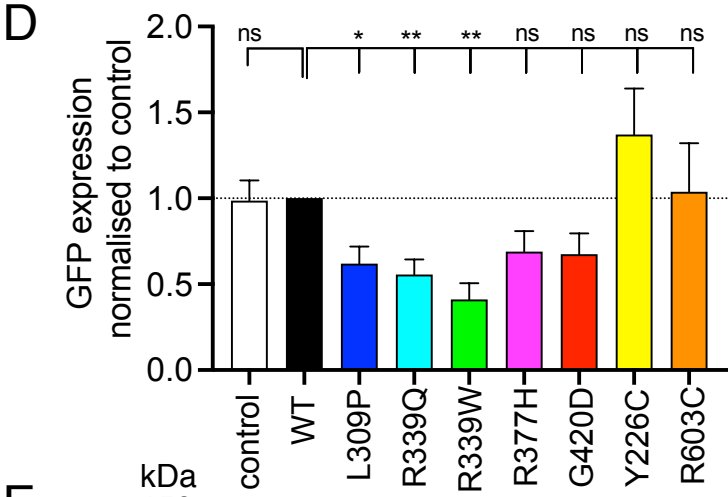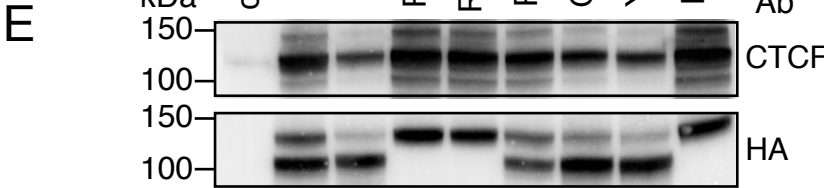

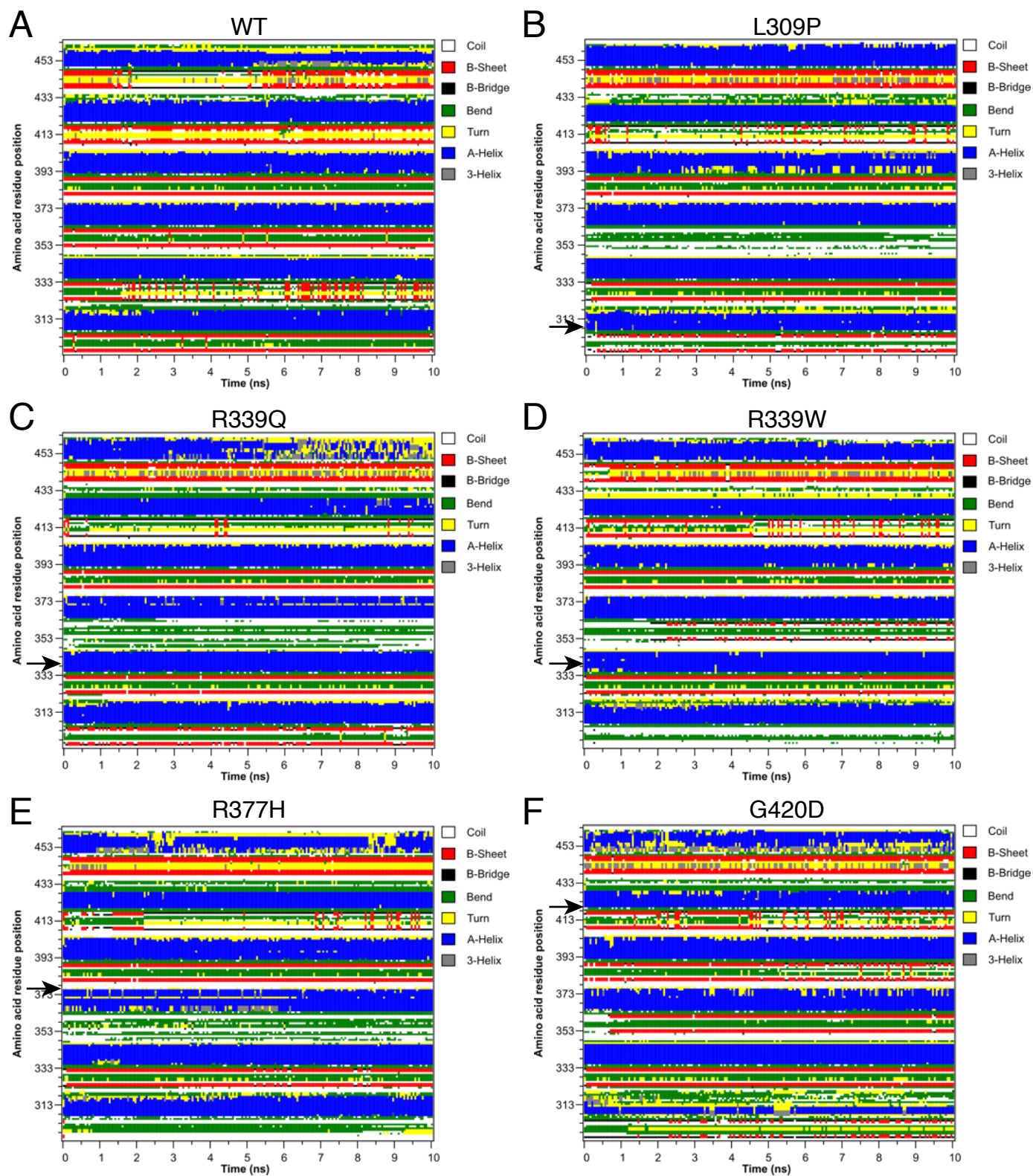

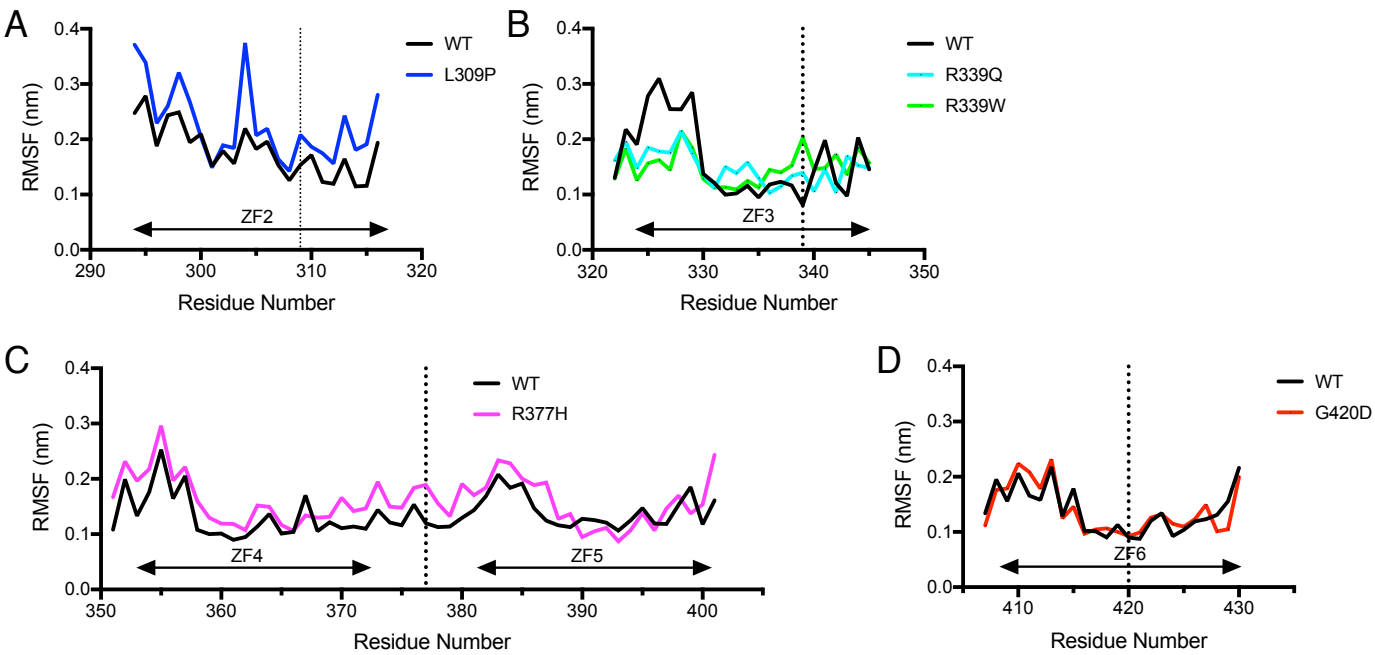

Supplement: Supplementary file 5 — Supplementary Figure 5 Flexibility of CTCF ZFs before and after mutation measured by MD simulations. (A-D) Backbone RMSF values during the MD simulations for mutant CTCF ZFs compared to WT, spanning their associated ZF domain; dotted vertical line indicates position of mutation. (A) L309P; ZF2 (B) R339Q & R339W; ZF3 (C) R377H; ZF4 & 5, (D) G420D; ZF6. (PDF 5317 KB) [file 18_2021_3946_MOESM5_ESM.pdf]
